# Incidence of solitary pulmonary nodules in Northeastern France: a population-based study in five regions

**DOI:** 10.1186/s12885-016-3029-z

**Published:** 2017-01-11

**Authors:** Émilie Marrer, Damien Jolly, Patrick Arveux, Catherine Lejeune, Marie-Christine Woronoff-Lemsi, Jérémie Jégu, Francis Guillemin, Michel Velten

**Affiliations:** 1 Department of Epidemiology and Public Health, Faculty of medicine, EA 3430, Strasbourg University, Strasbourg, France; 2 Clinical research Coordination, University Hospital, Reims, France; 3 Reims Champagne-Ardenne University, EA 3797, Reims, France; 4 Medical Information Department, Centre Georges-François Leclerc, Dijon, France; 5 Institut national de la santé et de la recherche médicale (INSERM), Unité 866, Faculty of Medicine, Dijon University, Dijon, France; 6 Besançon University Hospital, Délégation à la Recherche Clinique et à l’Innovation, Place Saint-Jacques, Besançon, France; 7 Franche-Comté University, EA 4267, Besançon, France; 8 Department of Public Health, University Hospital of Strasbourg, Strasbourg, France; 9 Nancy-University, EA 4360 Apemac, Nancy, France; 10 Institut National de la Santé et de la Recherche Médicale (INSERM), Centre d’Investigation Clinique – Épidémiologie Clinique, Nancy University Hospital, Nancy, France; 11 Department of Epidemiology and Biostatistics, Centre Paul Strauss, Strasbourg, France

**Keywords:** Solitary pulmonary nodule, Incidence, France, CT scan, MRI imaging, Lung cancer

## Abstract

**Background:**

The discovery of a solitary pulmonary nodule (SPN) on a chest imaging exam is of major clinical concern. However, the incidence rates of SPNs in a general population have not been estimated. The objective of this study was to provide incidence estimates of SPNs in a general population in 5 northeastern regions of France.

**Methods:**

This population-based study was undertaken in 5 regions of northeastern France in May 2002-March 2003 and May 2004-June 2005. SPNs were identified by chest CT reports collected from all radiology centres in the study area by trained readers using a standardised procedure. All reports for patients at least 18 years old, without a previous history of cancer and showing an SPN between 1 and 3 cm, were included.

**Results:**

A total of 11,705 and 20,075 chest CT reports were collected for the 2002–2003 and 2004–2005 periods, respectively. Among them, 154 and 297 reports showing a SPN were included, respectively for each period. The age-standardised incidence rate (IR) was 10.2 per 100,000 person-years (95% confidence interval 8.5–11.9) for 2002–2003 and 12.6 (11.0–14.2) for 2004–2005. From 2002 to 2005, the age-standardised IR evolved for men from 16.4 (13.2–19.6) to 17.7 (15.0–20.4) and for women from 4.9 (3.2–6.6) to 8.2 (6.4–10.0). In multivariate Poisson regression analysis, gender, age, region and period were significantly associated with incidence variation.

**Conclusions:**

This study provides reference incidence rates of SPN in France. Incidence was higher for men than women, increased with age for both gender and with time for women. Trends in smoking prevalence and improvement in radiological equipment may be related to incidence variations.

## Background

The discovery of a solitary pulmonary nodule (SPN) on a chest imaging exam has been of major clinical concern since the 1950s and has become common in current clinical practice since the widespread use of computed tomography (CT) [1–3]. Despite several studies on the frequency of SPN discovered on imaging exams, incidence data (number of new cases of SPN occurring within a specified period of time among person-time at risk in the population) are still scarce, especially in a general population. In the 1950s, one SPN was found for every 500 to 1000 chest radiographs in the USA, depending on the population studied [4–6]. Since the 1990s, CT screening programs for lung cancer have been providing estimates of the prevalence of non-calcified nodules discovered in high-risk participants, but data on the incidence of nodules, and especially SPNs, are neither detailed enough nor even provided [7–11]. Furthermore, they relate to groups at-risk and not to the general population. Recently, a study among the Kaiser Permanente Southern California member population estimated that the incidence of incidental pulmonary nodules of size 4–30 mm increased from 4.7 per 1,000 in 2008 to 5.4 in 2012 [12]. Despite the epidemiological and clinical relevance of these estimations, authors did not distinguish SPNs from multiple nodules.

The lack of incidence data for SPNs may be explained by the difficulty in obtaining non-biased data. Indeed, SPNs represent multiple pathological entities, and their assessment does not necessarily require a hospital stay [13]. Therefore, unless a registration system of ambulatory care diagnosis has been set up, common sources of medical information, such as health insurance or hospital discharge data in France, fail to identify all cases. Moreover, another difficulty in the identification of incident cases is to ensure that the presence of the nodule has not been noticed before. Nevertheless, incidence data - measuring the speed of occurrence of a pathology in the population - are useful to determine the importance of this pathological entity in daily clinical practice and to estimate human and material health care resources needed. Finally, as the epidemiology of pulmonary nodules evolves with imaging practices, a point estimate of SPN incidence is essential to analyse future trends [12].

This study aimed to estimate incidence rates of SPNs in a general population in 5 northeastern regions of France for two periods and to identify factors associated with differences in incidence, using data collected within the framework of a medico-economic evaluation program.

## Methods

### Study design and setting

This population-based study was undertaken in 5 contiguous northeastern regions of France (Alsace, Bourgogne, Champagne-Ardenne, Franche-Comté and Lorraine) comprising 8,200,000 inhabitants and representing about 13% of the French population. This study is part of a larger medico-economic evaluation program that took place from May 2002 to March 2003 and from May 2004 to June 2005. This program was set up to analyse the consequences of the implementation of positron emission tomography (PET) cameras in France on the diagnostic and therapeutic management of SPN and on other clinical situations in oncology [14].

### Participants

New SPN cases were identified from the analysis of CT reports including the thorax and performed for various reasons in current clinical practice. As a CT exam could also include abdomen and pelvis besides thorax, these reasons could be either pulmonary or non-pulmonary. All radiological centres performing chest CT imaging (community centres, as well as teaching, public or private centres) participated for the 2 periods of the study. Chest CT reports were collected in each centre during 4 weeks (Lorraine) or 6 weeks (other regions) in 2002–2003 and during 8 weeks in all regions in 2004–2005. The subperiods of data collection were randomly assigned to each centre over the calendar year to cover a continuous period of 10 and 15 months for each study period, respectively. Collection of CT reports was exhaustive over the designated subperiods in each centre.

Reports for patients at least 18 years old with SPNs between 1 and 3 cm were included. Exclusion criteria were: a nodule already known, reports for patients with a current or past history of cancer (non-melanomatous carcinomas excepted), a diagnostic procedure not performed in the study area, spiculated or calcified SPN seen on chest radiography and mentioned as such on the CT report, multiple nodules, or ground glass opacities.

### SPN report inclusion and data collection

In each region, a clinical research associate (CRA) was in charge of collecting and analysing all reports of chest CT. During ad-hoc sessions, the study investigators trained CRAs to read the CT reports to ensure homogeneous results. An audit was carried out during each study period to verify that the methods used by the CRAs to collect data were in accordance with the operative procedures defined in the reference manual, which contributed to the homogeneity of the results.

A CT report was eligible if it mentioned terms suggesting the presence of a SPN (“nodule”, “spherical lesion”, “round opacity”, “consolidation” etc.). For each eligible report, information about a pre-existent nodule was sought on the CT report, and checked in medical records or by contact with the patient’s general practitioner to ensure that the presence of the nodule had not been noticed before. Only new cases were considered as incident.

After CRAs selected a first round of eligible reports in each region, all reports were pooled and reviewed by the 5 CRAs together. A report was included or excluded if at least 4 CRAs agreed. Reports without agreement were then reviewed by a panel of 5 physicians. These reports were included or excluded if all physicians agreed. Finally, reports remaining without agreement were reviewed by the steering committee composed of the 5 physicians and the 5 CRAs who made the final decision on inclusion. The inclusion procedure was identical for both periods of the study.

The CRAs collected data on age, gender, history of cancer and smoking, the referring physician for chest CT, size category (1-3 cm versus < 1 or > 3 cm) and appearance (calcified or spiculated) of the nodule seen on CT, and whether the SPN was discovered by chest radiography or CT. They collected information on potential development of lung cancer and vital status by telephone contact with the patient’s physician 6 months after the chest CT was performed.

### Statistical analysis

Annual French population data were provided by the Institut national de la statistique et des études économiques (INSEE).

For each study period, crude incidence rates were estimated by gender, age group, region and for the whole area covered by the 5 regions, by dividing the number of new SPN cases by the sum of the person-times at risk in the population over the study period. Incidence rates were standardised to the world reference population [15] by the direct method (to enable international comparisons). Estimations were based on the hypothesis of a stationary population and a stable incidence rate of SPN during the 2002-2005 period.

For crude and age-specific incidence rates, 95% confidence intervals (95% CIs) were determined by the Poisson distribution. For standardised incidence rates, because usual conditions were met, a normal approximation was applied [16]. The binomial distribution was used to determine 95% CIs for proportions. Fisher’s exact test was used to compare the distribution of cancer cases according to gender, period and histologic subtypes. Poisson regression models including gender, region, age-class and period were created to identify factors related to variations in incidence rates (dummy variables were used as appropriate). None of the interactions included in the model was significant except for the interaction between region and period. Thus, a stratified Poisson regression model according to the period was built. The SAS 9.2 statistical software was used for the statistical analyses (SAS Inst., Cary, NC, USA).

The study protocol was approved by the institutional review board in France, the Commission Nationale de l’Informatique et des Libertés (CNIL) on January 2002.

## Results

### Participants

**Fig. 1:**
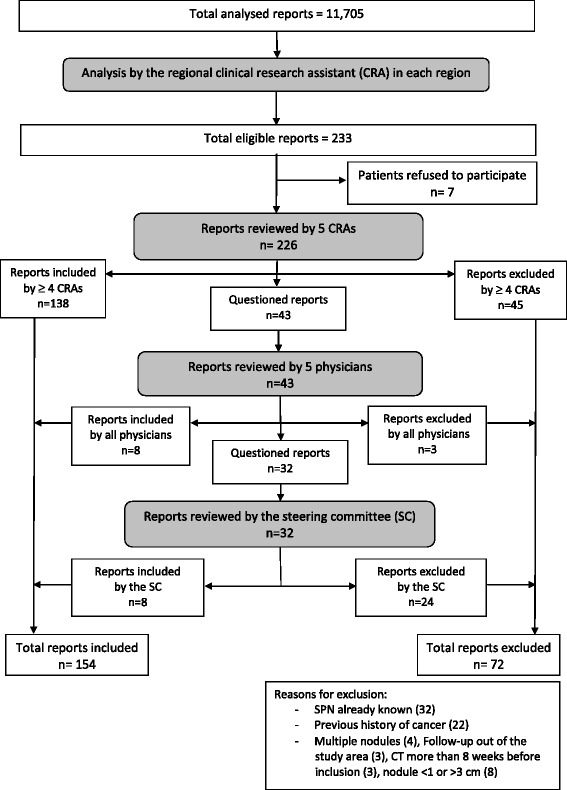
Flow-chart of CT reports analysed in 2002–2003

**Fig. 2:**
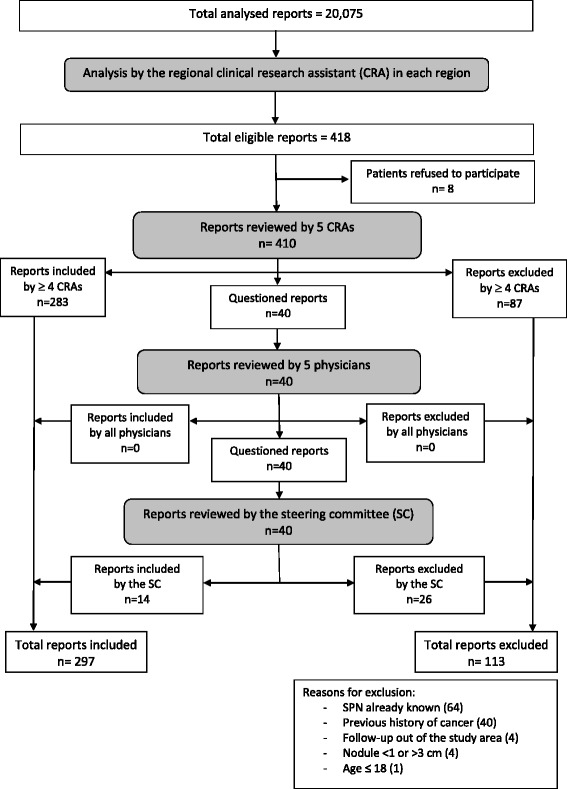
Flow-chart of CT reports analysed in 2004–2005

A total of 11,705 and 20,075 chest CT reports were analysed for the 2002–2003 and 2004–2005 periods, respectively (Figs. 1 and 2). Overall, 233 (2.0%) and 418 (2.1%) reports, respectively, were considered eligible. After exclusion of reports that did not meet the inclusion criteria, 154 (1.3%) and 297 (1.5%) reports, respectively, were included. Information about lung cancer incidence and vital status at 6 months was collected for 125 (81%) and 240 (81%) patients, respectively.

### Description of patients

Mean age (SD) at diagnosis was 65 (14) years (range 31–92) in 2002–2003 and 64 (14) (range 22–90) in 2004–2005. Overall, 73% and 65% were men for each period, respectively (Table 1). Among subjects whose smoking status was known (76% and 80%, respectively), 82% and 65% were current or ex-smokers, respectively. For both periods, the chest CT was prescribed by a specialised physician in approximately 70% of cases, otherwise by a general practitioner. The presence of the SPN’s characteristic spiculated or calcified was notified on chest CT reports for less than half of the subjects (Table 1). In 2002–2003, nodules were discovered equally by chest radiography or CT, whereas CT was predominant in 2004–2005 (Table 1).

**Table 1:** Characteristics of patients and nodules

	2002–2003		2004–2005	
	*n* = 154		*n* = 297	
	n	%	n	%
Patients				
Age				
≥ 50 years	127	82.5	245	82.5
Gender				
Men	112	72.7	193	65.0
Women	42	27.3	104	35.0
Region				
Alsace	32	20.8	79	26.6
Bourgogne	18	11.7	36	12.1
Champagne-Ardenne	32	20.8	43	14.5
Franche-Comté	24	15.6	27	9.1
Lorraine	48	31.1	112	37.7
Smoking				
Current or ex-smokers	96	82.1	154	64.7
Non smokers	21	17.9	84	35.3
No information	37		59	
Referring physician for CT				
General practitioner	41	29.1	89	30.0
Specialist	100	70.9	208	70.0
No information	13		0	
Solitary pulmonary nodule				
Spiculation				
Yes	49	81.7	55	37.9
No	11	18.3	90	62.1
No information	94		152	
Calcification				
Yes	13	33.3	54	36.5
No	26	66.7	94	63.5
No information	115		149	
Discovered by				
Chest radiography	79	53.4	101	35.2
Chest CT	69	46.6	186	64.8
No information	6		10	
Follow-up at 6 months				
Cancer				
Yes	33	26.4	53	22.1
No	92	73.6	187	77.9
No information	29		57	
Death				
Yes	24	15.6	26	8.8
No	130	84.4	271	91.2

### Incidence rates

**Fig. 3:**
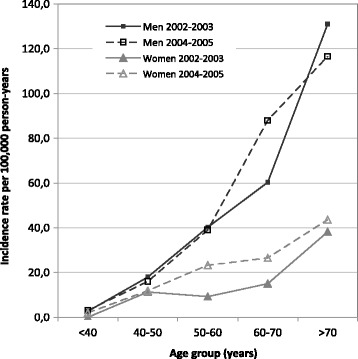
Incidence rates of SPNs for 5 regions in northeastern France for 2002-2003 and 2004-2005 by gender and age

The world age-standardised incidence rate was 10.2 per 100,000 person-years (95% CI 8.5–11.9) for 2002–2003 and 12.6 (11.0–14.2) for 2004–2005. Between 2002 and 2005, age-standardised rates evolved from 16.4 (13.2–19.6) to 17.7 (15.0–20.4) for men and from 4.9 (3.2–6.6) to 8.2 (6.4–10.0) for women (Table 2). For men, crude incidence rates were 26.9 (22.1–32.3) new cases of SPN per 100,000 person-years in 2002–2003 and 28.2 (24.4–32.5) in 2004–2005. For women, crude rates were 9.6 (6.9–13.0) and 14.5 (11.9–17.6), respectively, for each period (Table 2). In both genders, incidence increased with age, and the increase was more marked in men than in women (Fig. 3).

**Table 2:** Interregional incidence rates per 100,000 person-years by age, gender and period

		Men								Women							
		2002–2003				2004–2005				2002–2003				2004–2005			
Person-years		416,926				683,299				437,200				716,751			
		IR^a^	95% CI^b^			IR^a^	95% CI^b^			IR^a^	95% CI^b^			IR^a^	95% CI^b^		
IR^a^ by age group (years)	20–25	0.0	0.0	–	10.8	0.0	0.0	–	6.5	0.0	0.0	–	11.1	2.3	0.1	–	12.6
	25–30	0.0	0.0	–	11.2	9.6	2.6	–	24.6	0.0	0.0	–	11.4	9.8	2.7	–	25.2
	30–35	6.6	0.8	–	23.7	6.2	1.3	–	18.2	0.0	0.0	–	10.0	2.1	0.1	–	11.7
	35–40	12.8	3.5	–	32.8	8.0	2.2	–	20.6	0.0	0.0	–	9.7	2.0	0.1	–	11.3
	40–45	13.0	3.5	–	33.3	5.9	1.2	–	17.4	19.2	7.1	–	41.9	7.9	2.1	–	20.1
	45–50	22.9	9.2	–	47.2	26.3	14.0	–	45.0	3.2	0.1	–	18.0	15.8	6.8	–	31.2
	50–55	45.3	24.7	–	75.9	30.3	17.0	–	50.0	9.8	2.0	–	28.7	24.1	12.5	–	42.1
	55–60	33.7	14.5	–	66.3	48.6	30.4	–	73.5	8.5	1.0	–	30.8	22.3	10.7	–	41.0
	60–65	53.6	25.7	–	98.5	86.0	56.1	–	125.9	15.4	3.2	–	45.1	35.3	17.6	–	63.2
	65–70	67.3	34.8	–	117.6	90.0	58.8	–	131.9	14.6	3.0	–	42.6	18.2	6.7	–	39.6
	70–75	136.7	85.7	–	207.0	114.1	77.0	–	162.9	52.6	26.3	–	94.2	23.6	10.2	–	46.6
	>75	127.1	85.1	–	182.5	118.3	86.9	–	157.3	31.0	16.5	–	53.0	53.0	37.5	–	72.8
Crude rate		26.9	22.1	–	32.3	28.2	24.4	–	32.5	9.6	6.9	–	13.0	14.5	11.9	–	17.6
Standardised IR^c^		16.4	13.2	–	19.6	17.7	15.0	–	20.4	4.9	3.2	–	6.6	8.2	6.4	–	10.0

^a^ *IR* incidence rate

^b^95% CI: 95% confidence interval (Poisson exact CI for IR by age group and crude rates, normal approximation for standardised IR)

^c^Standardised IR: on the world reference population [15]

In multivariate analysis, no significant interaction between gender and period was evidenced (*p* = 0.10). However, when a Poisson regression model was stratified according to gender, the period effect (2004–2005 vs 2002–2003) adjusted for age and region was significant in women (incidence rate ratio [IRR] = 1.53, 95% CI [1.07–2.19]) but not in men (IRR = 1.07, 95%CI [0.84–1.34]). In both genders, incidence rates were significantly related to age (*p* < 0.0001), but men aged 70 years or more compared to men aged 50–59 years had a higher IRR than their female counterparts (3.50 [2.25–5.44] vs 2.34 [1.50–3.64]).

Incidence also fluctuated among regions and on the whole, incidence rates and IRR were higher in Alsace, Champagne-Ardenne and Lorraine than in Bourgogne and Franche-Comté (Tables 3 and 5). In multivariate analysis, incidence rates were significantly related to gender, age, region and period by Poisson regression. A significant qualitative interaction between region and period was identified (*p* = 0.004). The estimated IRR for men versus women was 3.47 (2.43–4.96) in 2002–2003 and 2.32 (1.83–2.95) in 2004–2005 (Table 4). For both periods, incidence rates were significantly related to age and region (Table 4).

**Table 3:** Incidence rates per 100,000 person-years by region, gender and period

		Alsace		Bourgogne		Champagne-Ardenne		Franche-Comté		Lorraine	
		IR^a^	95% CI^b^	IR^a^	95% CI^b^	IR^a^	95% CI^b^	IR^a^	95% CI^b^	IR^a^	95% CI^b^
Men											
2002–2003	Crude rate	22.0	13.8–33.3	12.2	6.1–21.8	29.2	18.3–44.3	26.5	15.4–42.4	45.9	32.8–62.5
	Standardised IR^c^	14.9	8.5–21.3	6.0	2.1–9.9	19.1	10.8–27.4	16.8	8.6–25.0	27.9	18.9–36.9
2004–2005	Crude rate	37.0	27.5–48.8	20.7	13.4–30.6	28.0	18.6–40.5	10.5	6.0–17.0	42.4	33.3–53.2
	Standardised IR^c^	24.3	17.3–31.3	12.9	7.6–18.2	17.1	10.2–24.0	6.3	3.0–9.6	27.5	20.9–34.1
Women											
2002–2003	Crude rate	9.6	4.6–17.6	7.3	2.9–15.0	12.7	6.1–23.3	10.5	4.2–21.7	8.8	3.8–17.3
	Standardised IR^c^	5.4	1.8–9.0	3.6	0.6–6.6	6.3	1.8–10.8	6.6	1.3–11.9	3.6	0.7–6.5
2004–2005	Crude rate	20.5	13.7–29.4	8.5	4.3–15.3	14.2	8.0–23.5	6.9	3.5–12.4	20.8	14.7–28.5
	Standardised IR^c^	12.1	7.1–17.1	3.7	1.0–6.4	8.5	3.4–13.6	3.8	1.1–6.5	12.4	8.0–16.8

^a^IR: incidence rate

^b^95% CI: 95% confidence interval (Poisson exact CI for IR by age group and crude rates, normal approximation for standardised IR)

^c^Standardised IR: on the world reference population [15]

**Table 4:** Effect of gender, age, and region for each study period estimated by stratified Poisson regression

	IRR^a^	95%CI^b^	*p*
2002–2003			
Gender			<0.0001
Men	3.47	2.43–4.96	
Women	1 (reference)		
Age			<0.0001
< 40	0.05	0.02–0.13	
40–49	0.59	0.32–1.06	
50–59	1 (reference)		
60–69	1.52	0.90–2.59	
≥ 70	3.50	2.25–5.44	
Region			0.0003
Alsace	1 (reference)		
Bourgogne	0.50	0.28–0.89	
Champagne-Ardenne	1.22	0.75–1.99	
Franche-Comté	1.07	0.63–1.81	
Lorraine	1.61	1.03–2.51	
2004–2005			
Gender			<0.0001
Men	2.32	1.83–2.95	
Women	1 (reference)		
Age			<0.0001
< 40	0.08	0.05–0.13	
40–49	0.44	0.28–0.69	
50–59	1 (reference)		
60–69	1.84	1.30–2.60	
≥ 70	2.60	1.91–3.56	
Region			<0.0001
Alsace	1 (reference)		
Bourgogne	0.42	0.28–0.62	
Champagne-Ardenne	0.68	0.47–0.99	
Franche-Comté	0.28	0.18–0.43	
Lorraine	1.03	0.77–1.38	

^a^IRR: Incidence rate ratio

^b^95% CI: 95% confidence interval

### Follow-up at 6 months

Six months after the inclusion of chest CT reports, 26% (19%–35%) and 22% (17%–28%) of patients with available follow-up information showed lung cancer, respectively for each period (Table 1). Lung cancer cases increased for women in 2004–2005, albeit not significantly (the ratio of males to females was 4.8 and 2.8 for 2002–2003 and 2004–2005, *p* = 0.44). Nodules in cancer patients were less calcified and more frequently discovered by chest radiography than CT. These patients were more often current or ex-smokers.

Squamous cell carcinoma represented the major type of lung cancer in each period (49 and 42%, respectively), followed by adenocarcinoma (36 and 34%, respectively). This distribution was similar between the 2 periods (*p* = 0.71) (Table 5). Among all SPN cases, 24 and 26 patients (16 and 9%), respectively, died within 6 months.

**Table 5:** Histologic subtypes of lung cancer

	2002–2003 (*n* = 33)			2004–2005 (*n* = 53)		
	n	%	95% CI^a^	n	%	95% CI^a^
Squamous cell carcinoma	16	48.5	41.2–55.8	22	41.5	36.2–47.1
Adenocarcinoma	12	36.4	29.6–43.7	18	34.0	28.9–39.5
Large-cell lung carcinoma	2	6.1	2.9–11.5	4	7.5	4.8–11.6
Small-cell lung carcinoma	1	3.0	0.9–8.0	2	3.8	1.8–7.3
Undetermined	2	6.1	2.9–11.5	7	13.2	9.7–17.8

^a^95% CI: 95% confidence interval

## Discussion

This population-based study provides incidence estimates of SPNs in a general population from 5 northeastern regions of France. Age-standardised incidence rates of SPNs were higher for men than women for the 2 periods and increased with age. Incidence also increased with time, significantly for women but not for men. As a comparison, the age-standardised incidence rate of SPNs in 2004–2005 was similar to that of bladder cancer in men (14.6) and ovarian cancer in women (8.1) [17].

Despite the lack of information on a possible relationship between smoking and the occurrence of pulmonary nodules, the higher incidence in men compared with women may reflect the more widespread use of tobacco among men. Indeed, although the prevalence of smoking has been increasing among women since the 1950s in France [18], until now, there were many more smokers among men than women [19]. Moreover, the incidence of lung cancer is higher for men than women [17]. As a result, clinicians might more easily prescribe a chest imaging exam such as a radiography or a CT for men than women, which might explain the higher detection rate of SPN in men. However, even if lung cancer incidence is higher for men than women, the incidence has been constantly increasing for women between 1980 and 2005 [17]. This trend could explain the significant increase in incidence in women between the two study periods. Indeed, the prescription of a chest CT for women could have been more frequent in 2005 than in 2002. However, in the recent study of Gould, incidence was slightly higher for women than men [12].

Several medical societies or physician working groups have produced guidelines on management strategies for incidental SPNs or non-small-cell lung cancer, but no consensus has been reached [20–26]. Nevertheless, worldwide and over time, a chest CT has been recommended to identify or characterize pulmonary nodule(s). Because no specific guidelines on SPN management exist in France, and because guidelines did not change between the two study periods, the increase in SPN incidence attributable to changes in medical practice is certainly small. Finally, because PET cannot be substituted for CT, the implementation of PET between the two periods certainly did not influence CT prescription practice. It is also unlikely that the implementation of PET would have impacted the evolution of SPN incidence rates.

However, after a delay in the development of medical imaging in France in comparison with other European countries such as Germany, the French government edited recommendations for the development of CT and magnetic resonance imaging (MRI) imaging in 2002 [27], that led to quantitative and qualitative improvement in the following years and could explain at least in part the increase in incidence of SPN we observed between these two periods. Indeed, from December 2003 by December 2005, the number of CT devices raised from 629 to 781 in France, representing an increase of 24% [28]. Despite no comparable data were available, neither for 2001 nor for 2002, we can deduce that the quantitative increase in CT equipment between the two study periods was superior to 25%.

In the same way, variations in human and material resources in medical imaging such as the density of radiologists or imaging devices could influence the prescription of such exams and partly account for the observed variability in incidence between regions. Actually by the end of 2003 and 2005, the two regions with the lowest SPN incidence rates, Bourgogne and Franche-Comté, had also the lowest density of radiologists, of CT devices and of imaging proceedings [29]. However, this could only partly account for the variation observed because Lorraine, for which the density of these indicators was also very low, yet had the highest SPN incidence rate.

Screening programs, despite providing recent valuable information, are limited in studying the incidence of SPNs. First, they target at-risk populations, mostly smokers aged 50 years and older. Second, the populations under study as well as the reporting of the results are fairly heterogeneous among studies. Indeed, incidence data for pulmonary nodules, especially SPNs, are not straightforward. Nevertheless, some data may be used to estimate the proportion of new, non-calcified nodules identified among all subjects screened by chest CT in 1 year. Thus, the incidence of non-calcified nodules was found to vary from 1% per year for new nodules ≥ 10 mm among smokers ≥ 60 years old in the New-York ELCAP study [10], to 2% per year for nodules > 5 mm in an Italian study of smokers ≥ 50 years [30]. In two other studies, the incidence rate of new non-calcified nodules ranged from 1.1 to 1.3% per year for smokers older than 50 [31, 32]. However, these proportions cannot be compared easily because screening programs recruit asymptomatic subjects, whereas in the present study, cases were recruited by current clinical practice procedures, which may involve both asymptomatic and symptomatic subjects referred to the radiologist. Finally, the study of Gould estimated that the incidence of incidental pulmonary nodules of size 4–30 mm increased from 2008 to 2012 [12]. However, although the source of CT exams was current clinical practice as in our study, the comparison is hampered by several factors: a larger nodule size definition, the identification of nodules on CT reports by a natural language processing algorithm that does not allow the distinction between solitary and multiple nodules, a different definition of incident nodules (no previous scan with pulmonary nodule(s) within the previous 2 years), and finally a different standard population used to report incidence data (US population 2010). These factors could partly explain the higher incidence reported in this Kaiser Permanente study.

The proportion of malignancies observed at 6 months was consistent with other published data. This probability varies considerably among studies, depending on the patient and nodule selection criteria (lung cancer risk factors, current clinical practice or surgical series) and the referral pattern of the study centre. In retrospective cohorts of SPN, it ranged from 23% (newly discovered SPN at the Mayo Clinic) to 58% (patients referred for fluorodeoxyglucose - positron emission tomography scanning) [33, 34]. In lung cancer screening studies, this proportion is weaker and varies from 3 to 21%, which is not surprising given that the underlying population is an asymptomatic one [7]. The proportion observed in the present study is intermediate, which is consistent with a recruitment from a general population subjected to current clinical procedures.

The distribution of histologic subtypes of lung cancer we observed was consistent with results of a French study showing that 40% of lung cancers were squamous cell carcinomas and 30% were adenocarcinomas, as well as with the results of an international study [35, 36].

One strength of the present study is that the collection of CT reports was exhaustive, standardised, and performed by specially trained CRAs. Moreover, this study was managed by an interregional steering committee. Although SPNs were included on the basis of CT reports, the number of SPNs discovered by another imaging exam that would not have led to a CT evaluation can be considered negligible. However, the SPN identification method based on the analysis of reports is less reliable than an analysis of radiological images.

Some limitations should be mentioned. First, the medico-economic evaluation program on which the present study was based focused on indeterminate nodules (for which malignancy is not foreseeable) because of the highest diagnostic benefit of PET in this category of nodules. Thus, subcentimetric nodules were not considered, as the probability of malignancy for these small nodules is much lower than for larger ones; consequently, clinical implications were quite different [23, 37]. In the same way, nodules that appeared spiculated or calcified on chest radiography, or ground-glass opacities, were excluded as the associated probability of malignancy is high. As a result, comparisons with other data should be made with caution.

Second, the population investigated was the general population subjected to current clinical practice procedures. As a result, subjects with asymptomatic SPNs who did not have any other reason to seek medical advice might not have been identified in the study. Indeed, these subjects may only be identified by a screening procedure. However, it would be ethically unacceptable to ask asymptomatic subjects, without any particular risk, to undergo CT or similar exams, only to ensure that no case of SPN would be missed in an epidemiologic study, given the uncertain benefits of such a procedure. Consequently, the incidence of SPNs can be considered slightly underestimated in this respect. Nevertheless, our study had the capacity to recruit subjects in a large variety of clinical situations and diagnostic pathways (symptomatic as well as asymptomatic, pulmonary as well as non-pulmonary, in the ambulatory setting as well as the framework of hospital care) and reasonably reflects the reality of daily clinical practice in northeastern France. Furthermore, we think that this point represents a strength rather than a weakness. However, detailed clinical indications for CT were not collected. This information would have been interesting to interpret the results because some infectious disease (particularly fungi infections) may increase the incidence of nodules.

Third, some data about smoking status and nodule characteristics could not be retrieved. Indeed, smoking status is not systematically mentioned on CT reports or in medical records, and is sometimes difficult to obtain retrospectively from the patient’s practitioners. Moreover, in the absence of clear guidelines concerning the reporting of imaging exams in France, CT reports are heterogeneous. However, we may be confident that a positive sign such as the presence of a calcification or a spiculation would always be mentioned on a CT report. Conversely, a negative sign, such as the absence of a radiological abnormality, might not be systematically mentioned. However, this limitation is not likely to significantly affect our results.

Finally, length of follow-up was only 6 months, which did not allow to determine the long-term frequency of subsequent cancer diagnosis. However, subjects for whom no information about a subsequent malignant evolution was available did not show any significant difference with respect to gender, age, spiculation or calcification of the nodule on CT, or vital status.

## Conclusions

This study provides reference incidence rates of SPN in the French general population. Incidence was higher for men than women and increased with age. Moreover, incidence increased between 2002 and 2005, significantly for women but not for men. These differences seem to reflect, at least in part, trends in tobacco consumption as well as improvement in radiological equipment during the study period.

## Abbreviations

95% CIs: 95% confidence intervals
CNIL: Commission Nationale de l’Informatique et des Libertés
CRA: Clinical research assistants’
CT: Computed tomography
INSEE: Institut national de la statistique et des études économiques
IRR: Incidence rate ratio
MRI: Magnetic resonance imaging
PET: Positron emission tomography
SD: Standard deviation
SPN: Solitary pulmonary nodule
